# Large-Scale Hybridisation as an Extinction Threat to the Suweon Treefrog (Hylidae: *Dryophytes suweonensis*)

**DOI:** 10.3390/ani10050764

**Published:** 2020-04-27

**Authors:** Amaël Borzée, Jonathan J. Fong, Hoa Quynh Nguyen, Yikweon Jang

**Affiliations:** 1 Laboratory of Animal Behaviour and Conservation, College of Biology and the Environment, Nanjing Forestry University, Nanjing 210037, China; amaelborzee@gmail.com; 2 Science Unit, Lingnan University, Tuen Mun, Hong Kong; 3 Department of Life Sciences and Division of EcoScience, Ewha Woman’s University, Seoul 03760, Korea; 4 Centre for Research and Development of Membrane Technology, Institute of Environmental Technology, Vietnam Academy of Science and Technology, Hanoi 10072, Vietnam

**Keywords:** hybridisation, extinction threat, North East Asia, hylid, conservation biology, Korea

## Abstract

**Simple Summary:**

A large number of amphibian species are now endangered, mostly because of human activities. An example is land modification, which may bring species that were previously isolated in contact, and allows them to hybridise. Here, we assessed the presence of hybrid individuals between the endangered Suweon treefrog (*Dryophytes suweonensis*) and the widespread Japanese treefrog (*Dryophytes japonicus*). We found hybrids to be relatively widespread and present at all populations where the Suweon treefrog occurred. This is important, as it results in an additional threat to the Suweon treefrog.

**Abstract:**

Amphibians are in the midst of a sixth mass extinction, and human activities play a major role in pushing species towards extinction. Landscape anthropisation has impacts that indirectly threaten species, in addition to the obvious destruction of natural habitats. For instance, land modification may bring human-commensal species in contact with sister-clades from which they were previously isolated. The species in these new contact zones are then able to hybridise to the point of reaching lineage fusion, through which the gene pool of the two species merges and one of the parental lineages becomes extirpated. Here, we documented the patterns of hybridisation between the spatially restricted *D. suweonensis* and the widespread *D. japonicus.* On the basis of the analysis of Cytochrome c oxidase subunit I mitochondrial DNA sequences (404 individuals from 35 sites) and six polymorphic microsatellites (381 individuals from 34 sites), we revealed a generalised, bi-directional, and geographically widespread hybridisation between the two species. Evidence of fertile back-crosses is provided by relatively high numbers of individuals in cyto-nuclear disequilibrium, as well as the presence of hybrid individuals further south than the species distribution limit, determined on the basis of call properties. Hybridisation is an additional threat to the endangered *D. suweonensis*.

## 1. Introduction

Threats to species are numerous, and most are linked to human activities [1,2,3]. For indirect threats such as hybridisation, anthropisation of the environment is often the catalyst that brings species into contact [4,5,6,7,8]. If two divergent species are brought back into contact and successfully reproduce to the point of collapsing into a hybrid swarm, the phenomenon is referred to as speciation reversal [9], albeit not a “reversal” per se, and is also called lineage fusion [10]. One example of reverse speciation due to human modification of the environment is whitefishes in Switzerland, where eutrophication due to human activities led to hybridisation and speciation reversal [11].

Although hybridisation in natural conditions can boost genetic diversity and allow for pre-speciation genetic variants to re-appear [12], it is generally geographically limited to contact zones, with alleles subject to strong selection held up at hybrid zones, and alleles that are not under selection “moving” through the hybrid populations [13]. In opposition, hybridisation caused by human activities is not necessarily restricted to contact zones, and often results in biodiversity loss [4,14]. The negative effects of hybridisation are outbreeding depression, decreased fitness, and, in extreme cases, the loss of a species and a reduction in biodiversity. In this last scenario, introgression erodes the genetic distinctiveness between species, until they collapse into a hybrid swarm where parents and hybrids can interbreed [11]. Such is the case between the mallard duck (*Anas platyrhynchos*) and New Zealand grey duck (*Anas superciliosa superciliosa*), where the latter species is under threat of extinction because of introgressive hybridisation [15,16]. An example involving amphibians is within the hybridogenetic *Pelophylax* complex in Europe, where the introduced species (*Pelophylax ridibundus*) hybridises with local species. Hybridisation is pushing the local species towards extinction, including *Pelophylax esculentus* and *Pelophylax lessonae* in Northern Europe [17], and *Pelophylax perezi* in Spain [18].

The Korean Peninsula is populated by two species of treefrogs: *Dryophytes suweonensis* [19,20] and *Dryophytes japonicus* [21,22]. The two species are genetically distinct [21,23,24,25], and differ in advertisement calls [19,26,27], morphometrics [19,28], and behaviour in relation to calling and microhabitat use [29,30]. The divergence between the “*D. japonicus* group” and the “*Dryophytes immaculatus* group” to which *D. suweonensis* belongs, is dated between 6.4 and 5.1 million years ago [31,32]. Currently, *D. japonicus* is the only species within the “*D. japonicus”* group [32] and *D. suweonensis* is the only species of the “*D. immaculatus* group” to occur on the Korean Peninsula [33].

The two species are sympatric, with the endangered *D. suweonensis* ranging from Mundeok in the Democratic People’s Republic of Korea [21,34,35,36] to Mankyeong River in the Republic of Korea [37,38]. To date, *D. suweonensis* has always been found syntopically with *D. japonicus* [38,39]. *D. japonicus* is distributed throughout most of Northeast Asia and is listed as “least concern” [40]. The two species are able to hybridise in laboratory conditions [41], but hybrids have so far not been documented in the wild.

Although the impact of hybridisation can be discussed in terms of threats or benefits to a species, the fact that the two focal species in this study have different karyotypes [42] will result in the loss of one or the other in the case of speciation reversal. In this study, we provide genetic evidence of hybridisation between the two species in the wild and highlight the potential role of human activities in their contact.

## 2. Materials and Methods

### 2.1. Field Work

**Figure 1:**
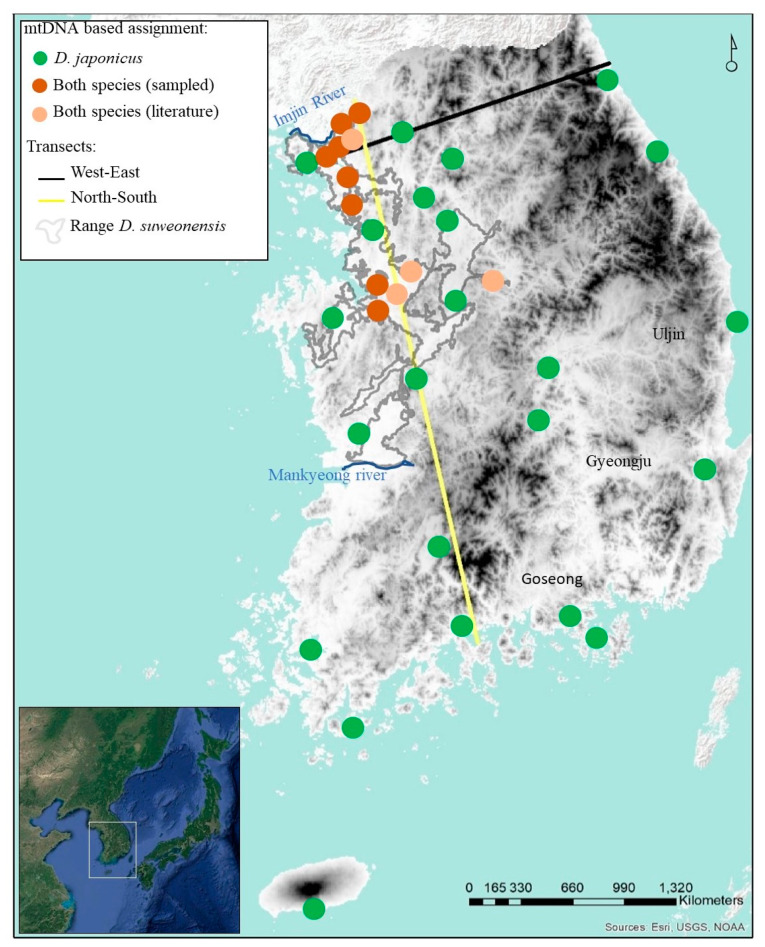
Sampling localities for all *Dryophytes* individuals in the Republic of Korea. Despite having sampled *Dryophytes suweonensis* only at some sites, later independent aural surveys indicated the presence of *Dryophytes japonicus* as well. Two transects across South Korea (west–east and north–south) were selected for subsequent genetic analyses on a subset of the samples (see details in Section 2.4 of the Materials and Methods section). Service layer credits: sources: Esri, United States Geological Surveys, and GeoServicesMap Esri Korea. Map generated in ArcMap 10.5 (Esri, Redlands, CA, USA).

When this project started in 2013, very little was known about the range and ecology of *Dryophytes suweonensis* [43,44]. Current data places the southern boundary of its distribution about 120 km north of the range described at the time of writing (Figure 1; [45]). As a result, sampling was conducted under blind conditions, aiming at the few known populations [39].

Field work was conducted following a rough grid with 20 km between sampled sites over the range of *D. suweonensis,* from what was known in 2013 [46]. Once beyond the estimated range of *D. suweonensis*, sampling sites were selected every 60 km to allow for a proper representation of the potential introgression patterns within the range of *D. japonicus*. Adjacent sampling sites were at least 12 km apart, ensuring independence of samples, as hylids disperse 2–4 km per year [47,48]. A total of 404 individuals from 35 sites (Figure 1) were collected, and genetic material was acquired through buccal swabs, frozen within 24 h at −20 °C [49]. The number of individuals sampled for each species was not determined during sampling, as the difference between species was not known until after sampling [28]. All work was completed under the authorisation of the South Korean Ministry of Environment (permit number 2013-16).

### 2.2. Molecular Work

DNA was extracted with the Enzynomics Genomic DNA Extraction Kit (Enzynomics, Daejon, Korea) following the recommendations of the manufacturer. All DNA samples were adjusted to 40 ng/μL before PCR reactions using a NanoDrop 1000 (Thermo Fisher Scientific; Waltham, MA, USA). PCR reactions targeting a 579 bp of mtDNA cytochrome c oxidase subunit I (COI) were performed on a PTC-100, BIO-RAD thermocycler (Bio-Rad Laboratories, Inc, California, CA USA) following the protocol of [26] using primers LepF1 (5′-ACC AAT CAT AAA GAT ATT GGT-3′) and LepR1 (5′-CCT CTG GGT GTC CGA AAA ATC A-3′). Samples were sent to Macrogen Inc. (Seoul, Korea) for direct sequencing, with both forward and reverse primers on an ABI PRISM 3100 automatic sequencer (Applied Biosystem Inc, Bedford, MA USA). We did not use additional sequences available from the National Center for Biotechnology Information as they were not from populations of interest [50].

A total of nine microsatellite markers developed by [51], and used by [42,52,53,54] were tested. After optimisation, only six of these polymorphic markers were successful in cross-amplifying the two target species (Table 1). As this project aimed at determining the presence and spread of hybridisation between the two *Dryophytes* species, and not to conduct a phylogenetic analysis, we considered the markers selected here to be adequate in order to accurately answer the question raised. This is supported by research reporting that as little as five microsatellite markers can be representative of the genetic structure of a population [55,56].

PCR reactions for all microsatellites contained the same volumes of reagent for a total of 20 μL but were run with different annealing temperatures (Table 1). We used the Takara PCR Buffer (initial concentration 10×, containing 2 mM MgCl_2_), dNTPs (10 mM), labelled primers (HEX or FAM; at 10 μM), and the Takara Taq polymerase (concentrated at 5 units/μL). The thermocycler (PTC-100, BIO-RAD; CA, USA) conditions used were 94 °C for 1 min, followed by 39 cycles at 94 °C for 30 s, with the specific annealing temperature for 1 min (Table 1), 72 °C for 1 min, followed by a terminal elongation at 72 °C for 5 min. PCR products were examined on a standard agarose gel at 1.5% before being sent for genotyping and allele scoring at Macrogen (Seoul, Republic of Korea). Allele scoring was verified using the microsatellites plug-in (Biomatters Limited, Auckland, New Zealand) in Geneious (v. 9.04, Biomatters Limited, Auckland, New Zealand).

### 2.3. mtDNA Analyses

Forward and reverse reads were assembled for each individual, checked by eye, and then aligned using the MUSCLE alignment plug-in in Geneious [57], with a maximum of 10 iterations. The sequences were first analysed through DnaSP [58], and 564 bp were used for analysis. We then used DnaSP [58] to compute haplotype diversity, Fu’s F, and Tajima’s D to test if the sequences selected were under selection or at mutation-drift equilibrium [59,60]. We then created a haplotype network in TCS with a fixed connection limit at 500 steps (estimated) and all other parameters set as defaults [61].

The relationship between the two species was inferred through the construction of phylogenetic trees using three different methods. For all trees, we used *Hyla chinensis* (GenBank accession number AY458593) as an outgroup, as *Hyla* is the closest related genus to *Dryophytes* [20]. First, we used neighbour-joining (NJ) to build a tree with the Geneious tree builder plug-in (Biomatters Ltd.), using a Jukes–Cantor genetic distance model. We performed 100,000 bootstrapping replicates to create a consensus tree with support at a 50% threshold. The second and third trees were generated using two different maximum likelihood (ML) algorithms (PhyML and RAxML). This duplicate analysis was conducted to assess the robustness of the tree topology resulting from different hill-climbing search methods, which potentially results in different tree scores [62]. The PhyML analysis was run through the PhyML plug-in for Geneious, using the GTR model (Lefort and Biomatters Ltd.; [63,64]). We chose an estimated gamma distribution parameter and used the combined subtree pruning and regrafting (SPR), plus nearest neighbour interchange (NNI) options for tree improvement. All other parameters were set as default, with optimisation for topology, branch length, and substitution rate. Bootstrap values were based on a 100,000-resampled dataset [63]. The RAxML analysis (Geneious plugin, [65]; RAxML v. 7.2.8) was run with a GTRGAMMA nucleotide model with 100,000 bootstrap replicates, and all other variables set to default [66].

### 2.4. Microsatellites analysis

We tested for the occurrence of null alleles or allelic dropout using a Bonferroni correction in Microchecker v. 2.2.3 (Norwich Research Park, Norfolk, UK [67]). We assessed the deviation from Hardy–Weinberg equilibrium with GENEPOP v. 4.2 (online access: https://genepop.curtin.edu.au; University of Montpellier, Montpellier, France [68]) through a *U*-test for each locus. We then calculated *F*-statistics on the basis of the six loci through FSTAT v. 2.9.3.2 (University of Lausanne, Lausanne, Switzerland [69]), with default parameters for all but two; we chose the rarefaction option to standardise the average estimated number of alleles per locus to remove the bias for sites with small sample sizes [70], and the Bonferroni corrections to address the problem of multiple comparisons.

**Figure 2:**
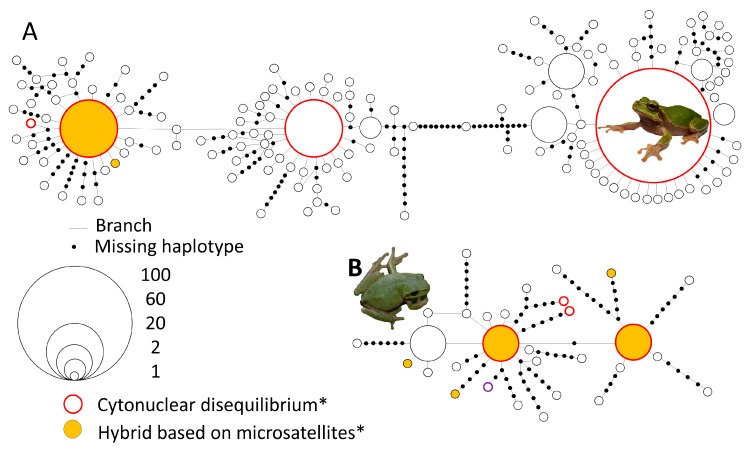
Haplotype network for **(A)** *Dryophytes japonicus* (*n* = 317) and **(B)** *Dryophytes suweonensis* (*n* = 87). The figure was drawn in TCS (v. 1.21 software: [61]) with a fixed connection limit at 500 steps (estimated) and all other parameters set as defaults. The star networks indicate pronounced population expansion (both species), whereas the missing haplotypes may be related to local extinctions among other possibilities such as inadequate sampling (*D. suweonensis*). *Denotes the presence of at least one individual in cytonuclear disequilibrium/hybrid for the haplotype highlighted.

We then used descriptive *F*-statistics to obtain the range of alleles sizes, the number of alleles, the allelic richness, and Nei’s estimation of observed and expected heterozygosity per locus [71]. The dataset was divided into three sub-sets on the basis of mtDNA: populations with *D. suweonensis* haplotypes only, *D. japonicus* haplotypes only, and populations with both haplotypes. Here, haplotypes defined the groups, as there was no overlap in haplotypes between the two clades (Figure 2). This ad-hoc analysis was conducted through tests based on 10,000 randomisations using F_IS_ statistics for Hardy–Weinberg equilibrium (HWE) within samples. SPSS v.21 (SPSS, Inc., Chicago, IL, USA) was used to statistically assess the variation between species.

For each locality with at least six individuals, we tested for recent bottlenecks with a one-tailed Wilcoxon test and Bottleneck v. 1.2.02 (INRA, Paris, France [72,73]). The minimum sample size was empirically determined and expected to be sufficient [74]. We tested for the presence of bottlenecks, as they would reflect significant decreases in population sizes and this way highlight the impact of external events, such as human activities, on population sizes. The parameters were set to the two-phase model (TPM) with 95% single-step mutations and 5% multiple-step mutations, run for 1000 iterations.

To evaluate the genetic structure of populations through the allelic content of haplotypes and frequencies, we performed analysis of molecular variance (AMOVA) among populations and individuals in IMa2 with 1000 permutations (Temple University, Philadelphia, PA, USA [75,76]). These analyses were executed for populations with a minimum of five individuals, as a low population size induces a bias in the analysis [77].

Patterns of genetic structure and the extent of hybridization between these two species were examined through the Bayesian clustering algorithm implemented in STRUCTURE v. 2.3.4 (University of Chicago, Chicago, USA) [78,79]. All analyses used data from the six microsatellite loci, and parameters were set to the default values provided by the program [79]. For each analysis, we tested a range of clusters (“K”) from one to the number of sampling sites plus three to determine the optimal value for “K”. The best fitting “K” was detected using the posterior probabilities (“LnP(D)”) obtained in the analyses and inferred from the method developed by [55], where ∆K is a summary statistic based on the rate of change of the likelihood distribution between successive values of K. The run with the highest log likelihood indicated the inferred number of populations within each species. We ran STRUCTURE 10 times for each K, with a burn-in of 70,000 steps followed by 700,000 MCMC steps [55,80,81], and averaged results for the 10 runs for each K. On the basis of these results, we determined the best fitting K for further analyses. To investigate the presence of hierarchical structure within the clades that cannot be detected by a one-step analysis [81], we also ran separate STRUCTURE analyses on “non-hybrid” mtDNA *D. suweonensis* and “non-hybrid” *D. japonicus* populations in order to test for cryptic intraspecific population structure and determine relationship between populations of the same clade. While doing so, we also ensured that the two groups were matching with the species assigned from mtDNA sequences.

Because of relatively similar values for the best fitting K defining the number of population clusters and the challenges in interpreting microsatellite data [82], we conducted additional analyses to interpret the population structure. As factorial dimension reduction through principal component analyses improves the description of the genetic structure and provides alternative assessment of the number of population clusters, we conducted a principal component analysis (PCA) on the basis of the genotyping results [83,84,85,86]. The number of population clusters, defined as significant principal components (PCs), can be determined using the distribution of the largest eigenvalue [83], resulting in the largest PCs being better correlated with genetic differences [85].

The PCA was based on the genotyping result for all 381 individuals, and was set so that principal components were extracted from all 12 variables (six markers; 2n) if their eigenvalue >1 under a varimax rotation (Table 2). Once the PCs were extracted, we tested for significance through a binary logistic regression with COI-defined clades as the dependent variable, and the PCs and locality as covariates. This test was conducted to confirm the K obtained from the STRUCTURE analysis, and thus determined the integrity of each clade considered here. The analysis was run under a main-effects model, and all assumptions were fulfilled—we did not detect any outlier when examining boxplots, and there was a linear relationship between the continuous independent variables and the logit transformation of the dependent variable, tested through the Box–Tidwell [87] procedure with Bonferroni corrections [88]. All analyses were conducted in SPSS (21.0; SPSS, Inc., Chicago, state, USA).

Once K was defined, the sites included in the analysis were reordered geographically from northwest to southeast along the coast of the Yellow Sea, and west to east over the estimated contact zone between the two species. We selected these transects as they cross the range of *D. suweonensis* along its two longest diagonals. The north–south transect was selected as it goes through the low plains of western Korea, a long and continuous landscape matching with the ecological requirements of the *D. suweonensis* [38,39]. We selected the west–east transect as it crosses the range of *D. suweonensis* at its widest point, along landscapes matching with the ecological requirements of the species, before crossing the Baekdudaegan mountain range where the focal species is not occurring as it is not found above altitudes of 100 m. To be included in a transect, a site had to be matching with the ecological requirements of *D. suweonensis*, as defined by [38,39]. The transects were used to test for the presence of a gradient in the hybridization pattern between the two clades.

### 2.5. Identification of Potential Hybrids

Introgression occurs when the genetic information of a clade is integrated into the genome of another following hybridisation and subsequent backcrossing [89], which results in tree incongruences when species are sympatric [90]. We identified hybrids using two methods. First, hybrid individuals were identified through their assignment to more than one clade through STRUCTURE, using Tq = 0.85 as threshold, meaning that any individual with less than 85% assignment probability to a clade was considered a hybrid. Tq is used to determine which clade an individual belongs to [91], and selecting a higher value can result in a parental taxa mis-identified as hybrid, whereas a lower value risks to misidentify an hybrid as a parental taxa. Traditionally it is set at 0.90 [78,92], although it is recommended that one selects a strict Tq value in order to determine the presence of hybrid with a higher certainty [91,93], and the use of Tq value ranges from 0.75 to 0.95 [92,94,95,96]. We followed the guideline provided by Vähä and Primer [92] (Figure 1) to select an adequate Tq for this study. Namely, because the F_ST_ value for all individuals together was above 0.21 and we used five loci, Tq = 0.8 may provide the best representative value, but Tq = 0.9 has a better efficiency. We decided to use an intermediate value (Tq = 0.85) to ensure representative hybrid assignment along with higher efficiency. In addition, a stricter value than Tq = 0.90 was required here to ensure we did not overestimate the threat of hybridisation to the species.

Second, we then assessed the presence of hybrids through cytonuclear disequilibrium, that is, inconsistency between mtDNA and nuclear assignment. An individual was considered a hybrid if assigned to one clade by STRUCTURE but to a mtDNA haplotype of the other clade [97]. Some hybrids were detected with both methods, whereas others were detected through microsatellite analyses only. Finally, cytonuclear disequilibrium was also analysed for directionality of hybridisation.

## 3. Results

### 3.1. mtDNA: Estimation of Genetic Variables

Sequences were recovered for all 404 individuals (GenBank accession numbers: MH181392-MH181792). The average number of pairwise nucleotide differences (k) was 26.31, and the nucleotide diversity (Pi) was 0.046. The total number of haplotypes (h) was 168, with a haplotype diversity (Hd) of 0.94 (variance <0.001). The separate analyses for the two species were based on 317 sequences for *Dryophytes japonicus* and 87 sequences for *D. suweonensis* (Figure 1). Further analysis for *D. japonicus* resulted in Tajima’s D = −2.38 (*p* > 0.05) and Fu’s F = −253.80, whereas Tajima’s D = −2.42 (*p* < 0.001) and Fu’s F = −29.35 for *D. suweonensis*. The negative and statistically significant D for *D. suweonensis* indicated an excess of rare mutations, potentially related to a population expansion, assuming the absence of selection. The negative Fu’s F for both species indicated an excess number of alleles and therefore population expansion, although the value related to population expansion was approximately nine times higher for *D. japonicus* than for *D. suweonensis*.

### 3.2. mtDNA Haplotype Network

The haplotype networks of the two species were not linked, and both species displayed clear structure (Figure 2). The network for *D. japonicus* included 317 individuals and was divided into three star-shaped sub-networks, with 18 missing haplotypes between two of the sub-networks (Figure 2A). The network for *D. suweonensis* included 87 individuals and was similar to *D. japonicus* in that there were two star-shaped sub-networks, although these were principally hollow. There were 15 missing haplotypes between the two most distance haplotypes (Figure 2B). The star-shaped subnetworks were smaller for *D. suweonensis* than *D. japonicus*, reflecting a less characteristic signature of population expansion.

### 3.3. mtDNA Analyses

**Figure 3:**
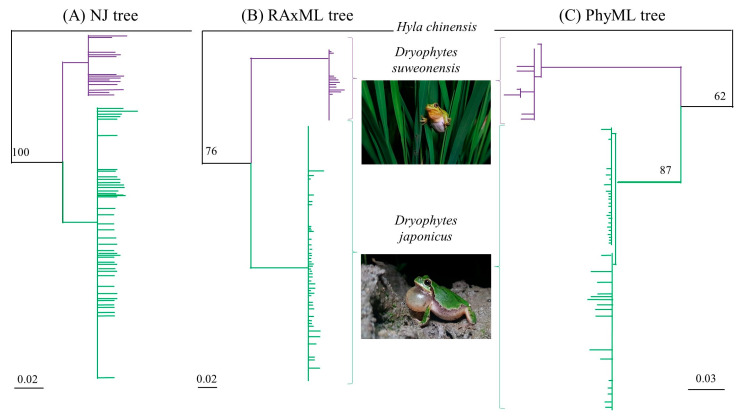
Mitochondrial phylogenetic trees inferred using (**A**) neighbour joining and maximum likelihood using (**B**) RAxML and (**C**) PhyML. All trees are built with *Hyla chinensis* (GenBank accession number AY458593.1) as outgroup. All trees are congruent, with the exception of a subclade within the PhyML tree as the only difference. All trees were built in Geneious (v 9.04, Biomatters Limited, Auckland, New Zealand) with corresponding plug-ins, and all nodes below 30% support were collapsed. Purple represents *Dryophytes suweonensis* and green represents *Dryophytes japonicus*. *Denotes the presence of at least one individual in cytonuclear disequilibrium/hybrid for the individual or collapsed clade highlighted.

On the basis of mtDNA, the three phylogenetic trees were congruent and the major clades (*D. suweonensis* and *D. japonicus*) were supported [98] (Figure 3). Sub-structuring was detected by PhyML (Figure 3C), bisecting the *D. japonicus* clade. This division of the *D. japonicus* clade was not detected by either the NJ (Figure 3A) or RaxML trees (Figure 3B).

The haplotype networks and phylogenetic trees agreed on the assignment of individuals to specific clades, with *D. suweonensis* detected at 12 out of 35 sampling localities (Figure 1). Within these 12 localities, 4 in the northern side of *D. suweonensis* range were devoid of *D. japonicus*, although sample sizes for these sites were low (3 < *n* < 13), and the species was detected at these sites in subsequent surveys [38].

### 3.4. Microsatellites Analysis

Genotyping was successful for 381 individuals from 34 sites. The analysis for the potential presence of null alleles identified a homozygote excess at WHA1-104. However, due to the low frequency of the two most numerous classes of alleles, which were 0.58 and 0.16 at five sites, we retained this location for further analysis. Furthermore, the analysis presented neither evidence of scoring error due to stuttering nor evidence for large allele dropout. None of the loci were detected as following HWE due to heterozygote deficit (SE = 0.001, switches = 1589.2, *p* > 0.001).

When looking at individuals assigned to either species through mtDNA and *F*-statistics, we detected low gene diversity for each locus when comparing expected and observed heterozygosity. The respective average values of 0.29 and 0.24 were found for sites harbouring *D. suweonensis* and *D. japonicus* only, whereas H_e_ at sites with both species was 0.28. The number of alleles per locus (A_e_) for sites with either species was low, but higher when both species were present. The patterns of allelic richness (AR) per locus were similar, with a mean of 1.27 alleles for sites with *D. suweonensis* only, 1.23 alleles for sites with *D. japonicus* only, and 1.40 for sites where both species were present (Table 3). The degree of inbreeding (F_IS_) was on average double for sites with *D. japonicus* only (0.62) compared with sites with *D. suweonensis* only (0.37), reflecting heterozygote deficiency. However, F_IS_ was not significantly different between averages for sites with one species compared to sites with both species (*T* = 0.853, *p* = 0.433).

The genetic differentiation within sites with *D. suweonensis* (F_ST_) was 0.22 on average, whereas a value of 1.19 within sites with *D. japonicus* indicated higher gene exchange between populations. The AMOVA among populations and individuals computed through IMa2 reported an estimated variation among sites of 0.45 (df = 3, *SS* = 332.20, *MS* = 14.44), an estimated variation among individuals of 0.67 (df = 319, *SS* = 546.04, *MS* = 1.72), and a variation within individuals of 0.37 (df = 343, *SS* = 128.5, *MS* = 0.375). The genetic variation among sites therefore accounted for approximately 30% of the total genetic variation, whereas the variation between individuals was approximately 45%. The second AMOVA between observed and random F_ST_-based distance matrices showed a negative correlation between genetic and geographical distances (*Rxy* = −0.074, *p* = 0.001). Finally, bottlenecks were detected for all *D. suweonensis* sites, and also for three *D. japonicus* sites.

The results of the analysis based on the pairwise F_ST_ values displayed a strong interspecies divergence (0.30, *p* < 0.01), but also the presence of two clusters within *D. japonicus* (0.28, *p* < 0.01), although these were not geographically organised. *Dryophytes suweonensis* was also divided in two clusters (0.35, *p* < 0.01), one north and one south of the city of Seoul and the Han River.

### 3.5. Population Structure

**Figure 4:**
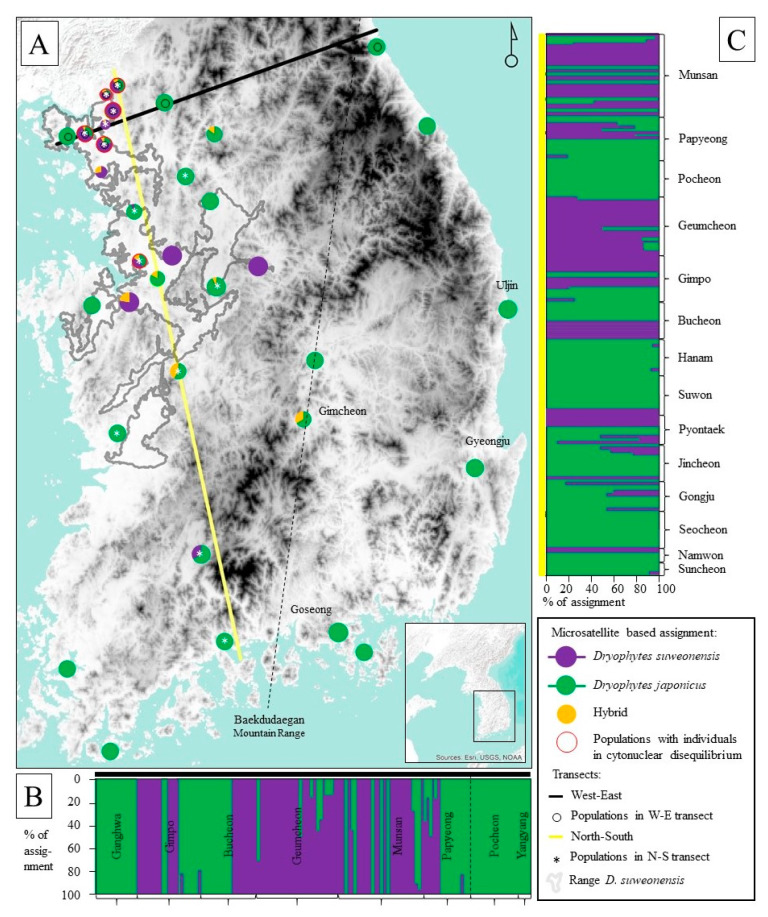
Structure analysis of microsatellite data, assigning each individual from populations selected for the transects to a clade based on percentage of assignment. (**A**) Pie charts represent the percentage of non-hybrid and hybrid individuals for each population, whereas the two transects ((**B**)—west–east; (**C**)–north–south; the names of localities are listed along the corresponding cardinal gradient and can be matched with the population following the annotated order on the map) represent individual assignments within populations. Structure assignments were drawn only if reaching the 10% threshold. Service layer credits: sources: Esri, USGS, and GeoServicesMap Esri Korea. Map generated in ArcMap 10.5 (Esri, Redlands, CA, USA).

The Bayesian analysis of population with STRUCTURE selected K = 2. The mean LnP(K) = 2786.41 for K = 1, mean LnP(K) = 3706.21 for K = 2, mean LnP(K) = 3573.13 for K = 3, and the mean LnP(K) = 3240.93 for K = 4. For K = 2, the mean value of F_ST_ for the two clades were 0.09 and 0.35, respectively. Out of 381 individuals, and based on a 15% minimal purity threshold, STRUCTURE assigned 125 individuals to *D. suweonensis*, 233 individuals to *D. japonicas*, and 23 individuals as hybrids (Figure 4A). No intraspecific substructure was detected within *D. japonicus* as the estimated Ln probability of data averaged over 10 runs was higher for “K = 1” (K = 1: 3013; K = 2: 2186; K = 3: 1854). However, results within *D. suweonensis* were less clear, with similar K value for K = 1 and K = 2 (K = 1: 1268; K = 2: 1246, K = 3: 976).

The PCA resulted in five PCs (Table 2), explaining 82.18% of total variation. Each gene fragment was principally loading in a different PC, with the exception of PC1. The model of the binary logistic regression was significant (Omnibus test; χ^2^ = 215.05, df = 6; *p* < 0.001) and explained 71.5% or variation (Nagelkerke *R^2^*). Contrasting with the STRUCTURE results, three out of the five PCs were significant, as well as location (Table 4). We decided to proceed with K = 2, from the STRUCTURE analysis, rather than K = 3 from the PCA because one of the *p*-values from the binary logistic regression is “barely” significant (*p* = 0.046; Table 4). Further analyses revealed the potential for a dichotomy within the *D. suweonensis* clade, but even if the clade was divided in two species, the question addressed in this manuscript regarding hybridisation between *D. japonicus* and the *D. suweonensis* clade would still be valid.

### 3.6. Identification of Hybrids

Comparing the STRUCTURE analysis and mtDNA results, we reported matching assignments for 234 individuals for *D. japonicus* and 68 for *D. suweonensis*. STRUCTURE-defined hybrid individuals were found at 12 sites (Figure 4), for a total of 23 individuals, among which 14 were assigned to *D. japonicus* and 9 to *D. suweonensis* on the basis of mtDNA (Figure 2 and Figure 3). All of these individuals were assigned to the two clades at varying degrees of confidence (Figure 4), therefore suggesting admixture. A total of 37 individuals exhibited cytonuclear disequilibrium, with 31 individuals assigned to *D. japonicus* through mtDNA and *D. suweonensis* through microsatellites, and six individuals with the reverse situation. No geographic pattern for individuals in cytonuclear disequilibrium was detected.

The STRUCTURE analysis for the west–east transect (*n* = 172) highlighted the absence of individuals in cytonuclear disequilibrium for non-hybrid *D. suweonensis* individuals east of the Baekdudaegan mountain range (Figure 4B). All non-hybrid *D. suweonensis* and hybrids were located in lowlands (Figure 4A). The STRUCTURE analysis for the north–south transect (*n* = 201) highlighted the presence of hybrid and non-hybrid *D. suweonensis* further south than the known range of the species on the basis of calls [38]. Hybrid individuals were identified across the whole range of *D. suweonensis* (Figure 4C). Finally, a hybrid individual with *D. japonicus* mtDNA was found east of the Baekdudaegan Range in Gimcheon.

## 4. Discussion

Our results demonstrate widespread hybridisation and bi-directional introgression between *D. japonicus* and *D. suweonensis* across the whole range of the latter species in the Republic of Korea. Consequently, *D. suweonensis*, an already declining species endemic to the Korean Peninsula [99], is under an additional threat. Evidence of this risk is highlighted by similar F_IS_ values between sites with either or both species, indicating that hybridisation rather than inbreeding is a major risk [100,101,102]. Hybridisation is also is supported by the relatively high number of individuals in cytonuclear disequilibrium, a result of bi-directional hybridisation followed by backcrossing [5,103]. It is possible that hybridisation partially results from human activities and especially local habitat modifications, regional habitat changes, and the creation of corridors [4]. We note, however, that incomplete lineage sorting can also affect STRUCTURE clustering, and this may affect the results presented here.

### 4.1. Genetic Structure

When assessing the match between biogeographical patterns, clade partitioning, and heterozygote deficit in our results, the genetic structure within both species corresponded to the anthropomorphised landscape features. In the case of *D. suweonensis*, the city of Seoul creates a permeable barrier between northern and southern populations, with two remote stepping stone populations likely resulting in interrupted gene flow [104]. This is likely important for *D. suweonensis*, as the species is not able to maintain population connectivity in the region, and this may explain the low difference between the values for K = 1 and K = 2 for the species. The similar values may reflect the ongoing segregation resulting from the loss of connectivity. We suspect, however, that we did not directly sample any population where the segregating clade was present, likely within the southern area of the species’ range, which would explain why the analyses based on mtDNA did not detect any variation. For *D. japonicus*, the lack of sub-clade structure presented by the NJ and RAxML trees may have pertained to the ability of the species to maintain species connectivity over the landscape, but it could also be the result of the less robust analysis in comparison to PhyML [105], as sub-clades in *D. japonicus* have been demonstrated [21,26]. This is supported by K = 1 from the STRUCTURE analysis of mtDNA-assigned *D. japonicus* individuals, as well as the lack of strong geographical structure in the two *D. japonicus* clades found on the basis of the F_ST_ matrix. We detected signatures of bottlenecks and F_ST_ values in *D. suweonensis* populations that were lower than the average for amphibians [48,52,106,107]. This likely reflected the fragmentation of the habitat [99,108] and the recent blockage of dispersal routes for potential gene flow [109].

### 4.2. Hybridisation

Bi-directional introgression seemed to be relatively higher for individuals assigned to *D. japonicus* (31 individuals) compared to *D. suweonensis* (6 individuals) when considering clade assignment through mtDNA. This unbalanced ratio was likely related to mating behaviour of calling males, and thus provided evidence for the threats to *D. suweonensis*. Male *D. suweonensis* call from the centre of rice paddies, whereas male *D. japonicus* call from the edges [29,30]. As female *D. suweonensis* approach breeding areas from the periphery and move toward the centre, they must cross a barricade of male *D. japonicus* to reach conspecific males. The same pattern has also been described in other treefrog species, such as *Dryophytes cinereus* and *Dryophytes gratiosus* [110]. As male anurans are undiscerning about the species they mate with [111,112], hybridisation is likely when they amplex non-conspecific females. In fact, male *D. japonicus* have been observed as being amplexed with several other species (*Rana amurensis*, *Rana uenoi*, *Hynobius leechii*, *Pelophylax nigromaculatus*, and *Pelophylax chosenicus*; author’s personal observations).

Hybrid individuals and individuals in cytonuclear disequilibrium were not clustered in sub-clades in the phylogenetic trees and haplotype network, highlighting the absence of genetic background for susceptibility to hybridisation. However, the presence of sites with hybrid individuals and individuals in cytonuclear disequilibrium was geographically structured—all sites were situated in the vicinity of the range of *D. suweonensis* (Figure 1 and Figure 4). This corresponded to the hypothesis suggesting that hybridisation is recent and that *D. japonicus* was not distributed on lowlands before landscape anthropisation because backcrossing over a large geographic area was not observed, and thus populations of *D. suweonensis* have been under hybridisation pressure since large scale landscape modification following the Korean War (1950–1953). The presence of individuals in cytonuclear disequilibrium outside the range of *D. suweonensis* also demonstrates the fact that backcrossing of hybrid individuals into the *D. japonicus* gene pool is not limited to the F1 generation. The north–south transect selected along the low plains of western Korea contrasted with the west–east gradient—the habitat is adequate for *D. suweonensis*, or at least for individuals resulting from hybridisation. Although hybrids are found further south than the range of *D. suweonensis*, no individuals in these southern regions exhibited the call properties of *D. suweonensis*—the Mankyeong River is the southern limit of individuals with these call properties [38]. A hybrid individual was found in Namwon, a locality east of the Baekdudaegan mountain range, but no individuals were identified as *D. suweonensis* on the basis of mtDNA, supporting backcrossing further than the F1 generation. This hypothesis is potentially supported by the increase in contact between the two clades through agriculture [113,114], which started circa 5000 BC [115]. The fact that no hybrid was found further east than the easternmost limit of *D. suweonensis* range at northern latitudes is unlikely to change with more data [38], and it shows that the species could not disperse further, likely because the habitat is not adequate [39], but also that hybrids inherit traits that prevent them from exploiting the habitat, contrary to non-*hybrid D. japonicus* individuals. This trait is likely to be related to the bolder behaviour of *D. japonicus* when dispersing [116]. It is, however, important to note that we used a 15% threshold to identify individuals as hybrid. A 10% threshold would result in the presence of four additional hybrid individuals, three of them far outside the range of *D. suweonensis*, in Goseong, Gyongju, and Uljin (Figure 4).

In conclusion, although it is not possible to demonstrate the origin of hybridisation with the data presented here, several markers point at three types of anthropisation of the habitat known to result in an increase in the probability of hybridisation [4]. These points are the fact that the species habitat underwent local habitat modifications [110,117,118,119], regional habitat changes allowed for the geographic expansion of a clade within the range of the other, and corridors were created leading to the continued movement of a clade within the breeding habitat of the other. Human activities started modifying the habitat circa 7000 years ago [115,120], with a strong development in the 1960s and 1970s in the form of tidal flat reclamation and rice paddy expansion [38]. As a result, the two species are now exploiting the hydroperiod of rice agriculture in a similar way, which brings the two species in the same habitat at the same time of the year [114]. Habitat modification also impacted connectivity between populations, and resulted in the fragmentation of the habitat used by D. suweonensis, but enabled D. japonicus to disperse comparatively further [26,39]. Therefore, it is difficult to exclude anthropisation of the habitat as one of the factors driving hybridisation between D. suweonensis and D. japonicus, along with hybridisation as an additional pressure on the already declining D. suweonensis.

## 5. Conclusions

In this study, we have demonstrated that Dryophytes suweonensis and Dryophytes japonicus hybridise, and that hybrid individuals were found in an area restricted to the vicinity of D. suweonensis’ range. The presence of individuals in cytonuclear disequilibrium highlighted the presence of fertile hybrids and events of backcrossing into both parental species. The microsatellite analysis also hinted at the potential presence of another clade that was not directly sampled in this study. We suggest that hybridisation between the two clades is related to the change in landscapes as a result of human activities, and especially because of the development of rice agriculture.

## Figures and Tables

**Table 1:** Microsatellite markers tested for cross-amplification in *Dryophytes japonicus* and *Dryophytes suweonensis.* The six microsatellite primer pairs for which cross-amplification was successful are in bold. These microsatellites were used to test hybridisation between Korean treefrogs.

Primer Name	Primer Sequence	Primer Developed/Used by	Annealing Temperature
**WHA1-9-F**	5′-CGTTTGGACGTGATGCTG-3′	Arens et al. [51] 2000, Arens et al. [52] 2006, Moreira [54] 2012	47
**WHA1-9-R**	5′-GAGGAGTTTCTTCACAAGGGG-3′		
**WHA5-201-F**	5′-TCATGGACTGTCGTCATGGT-3′	Arens et al. [51] 2000, Arens et al. [52] 2006, Moreira [54] 2012	47
**WHA5-201-R**	5′-AGGTAAATGGAATCTGGGTGTG-3′		
**WHA5-22A-F**	5′-TTACAGCAACAGCAAATGG-3′	Arens et al. [51] 2000, Arens et al. [52] 2006, Moreira [54] 2012, Stöck et al. 2011, Dusfresnes et al. 2015	50
**WHA5-22A-R**	5′-ATCAGGGACTGGGTCTGT-3′		
**WHA1-104-F**	5′-ACTTGGGACAGCCAGTATGTTTT-3′	Arens et al. [51] 2000, Arens et al. [52] 2006, Moreira [54] 2012	47
**WHA1-104-R**	5′-TGAGCTGGTGGGTATAACCTAAC-3′		
**WHA1-25-F**	5′-AAGAATCTGCCGCAAAGAAG-3′	Arens et al. [51] 2000, Arens et al. [52] 2006, Moreira [54] 2012	47
**WHA1-25-R**	5′-TAGGAAGGGACAGGAGGTCA-3′		
WHA5-57-F	5′-TTGTCCTGACATGCACACCT-3′	Arens et al. [51] 2000, Moreira [54] 2012	
WHA5-57-R	5′-CGTGTCTAACCCCAGCTCAT-3′		
**WHA1-140-F**	5′-ATGTGCCATAGAAATGAAGG-3′	Arens et al. [51] 2000, Arens et al. [52] 2006, Moreira [54] 2012	47
**WHA1-140-R**	5′-AGGCTTGCTGCTATTATGTC-3′		
WHA1-60-F	5′-TAGGTCATGTATAGCCTGTT-3′	Arens et al. [51] 2000, Arens et al. [52] 2006	47
WHA1-60-R	5′-TCTGTTTACTTCAGGGGT-3′		
WHA1-20-F	5′-GTCCCTTCCTGAATAAGTGTCG-3′	Arens et al. [51] 2000, Arens et al. [52] 2006	47
WHA1-20-R	5′-CCATTCCCTCCTGGCTTT-3′		

**Table 2:** Principal components extracted from a principal component analysis to determine clustering of genotyping data for *Dryophytes* sp. in the Republic of Korea. The variables were based on the genotyping result for 381 individuals and set so that principal components were extracted from all 12 variables (six markers; 2n) if their eigenvalue was >1 under a varimax rotation. Values with the highest loading factor are highlighted in bold. Please note that WHA1-104-R loads only weakly in any of the principal components (PCs).

Variables	PC1	PC2	PC3	PC4	PC5
WHA1-9-F	**0.87**	0.07	−0.08	0.00	0.04
WHA1-9-R	**0.86**	0.04	−0.03	0.04	0.09
WHA5-201-F	−0.09	0.01	**0.99**	0.00	0.03
WHA5-201-R	−0.09	0.01	**0.99**	−0.01	0.03
WHA5-22A-F	0.04	−0.01	0.02	0.04	**0.87**
WHA5-22A-R	−0.07	0.03	0.00	−0.22	**0.82**
WHA1-104-F	**0.71**	0.27	−0.08	0.10	−0.35
WHA1-104-R	0.36	0.24	−0.10	0.08	−0.32
WHA1-25-F	0.07	0.11	0.00	**0.96**	−0.09
WHA1-25-R	0.04	0.13	0.00	**0.96**	−0.10
WHA1-140-F	0.15	**0.97**	0.02	0.12	−0.01
WHA1-140-R	0.15	**0.97**	0.02	0.12	−0.01
Eigenvalues	3.28	2.09	1.78	1.45	1.26
% of variance	27.32	17.42	14.86	12.12	10.47

**Table 3:** *F*-statistics analyses averaged for all six loci. *p*-values are based on *t*-tests for difference between *Dryophytes japonicus* (*Dj*) and *Dryophytes suweonensis* (*Ds*) in Korea.

*F*-Statistics Analyses	All Populations	*Dj* Populations	*Ds* Populations	*p*-Value
Gene diversity (He)	0.28	0.246	0.29	0.746
Number of alleles (Ae)	2.564	2.12	2.167	0.928
Allelic richness (AR)	1.267	1.231	1.281	0.700
Inbreeding (F_IS_)	0.576	0.62	0.369	0.251

**Table 4:** Results of the binary logistic regression used to determine the number of independent genetic clusters. The analysis was run with cytochrome c oxidase subunit I (COI)-defined clades as the dependent variable and the principal components and locality as covariates. B is the unstandardized regression weight, SE the standard error and df the degree of freedom.

Binary Logistic Regression	B	SE	df	*p*-Value
PC1	−6.87	1.34	1	**<0.001**
PC2	15.94	4.26	1	**0.046**
PC3	4.61	1.42	1	0.211
PC4	−5.76	1.81	1	**<0.001**
PC5	1.82	0.74	1	0.294
Sampling locality	−0.05	0.03	1	**<0.001**
